# Socio-economic and cultural vulnerabilities to cervical cancer and challenges faced by patients attending care at Tikur Anbessa Hospital: a cross sectional and qualitative study

**DOI:** 10.1186/s12905-015-0231-0

**Published:** 2015-09-16

**Authors:** Sara Kebede Tadesse

**Affiliations:** Center for Gender Studies, Addis Ababa University, Addis Ababa, Ethiopia

## Abstract

**Background:**

Cervical cancer is a leading cause of death among women in Ethiopia, affecting them at a time of their life when they are critical to social and economic stability. This study was mainly focused on assessing different socioeconomic and cultural related factors that make women vulnerable to cervical cancer and challenges women face in the process of diagnosis and treatment.

**Methods:**

To achieve the objective of the study both qualitative and quantitative methods were utilized.198 participants were enrolled in a cross- sectional survey and 10 in-depth interviews were conducted with patients in Tikur Anbessa Hospital. A consecutive sampling method was used to select participants in the survey while purposive sampling was employed to select participants in the qualitative design.

**Results:**

For the population covered in the study, poverty along with other socio-cultural practices such as early marriage, high parity and to certain extent polygamy were identified as factors that increased the vulnerability of women to cervical cancer. In addition, the study has uncovered several challenges faced by cervical cancer patients in the diagnosis and treatment process. Three types of challenges that include, health care based, psychological and economic were identified. System and practitioner delay were found as the main hurdles within the variable of health care related challenges. What’s more, the psychological challenges identified included, fear of recurrence, negative social attitude and distress associated with the side effects from treatments such as fecal & urinary leakage. Furthermore, inability of bearing costs related to treatment and accommodation were cited as the main economic challenges.

**Conclusion:**

The study showed various socio-economic and cultural vulnerabilities that expose women to cervical cancer and the challenges encountered by cervical cancer patients after their diagnosis. Addressing this issue largely lies in strengthening primary and secondary preventive mechanisms, providing public education about safer sex practices, family planning and eliminating cultural practices such as early marriage and polygamy in connection to cervical cancer. Furthermore, improving the diagnostic and treatment procedures and facilities is also a crucial component that needs due emphasis in the fight against cervical cancer.

## Background

In recent years cervical cancer has become one of the most alarming health concerns in the world. Though cervical cancer remains a global issue, it mainly affects women in low resource areas. The incidence rate of cervical cancer in developed countries has been decreasing dramatically while it remains high in developing countries [1].

Cervical cancer is the second most common cancer in women worldwide and the leading cause of cancer deaths in developing countries. The 2010 published World Health Organization (WHO) report showed that out of 529,409 diagnosed women, 274,883 die annually from cervical cancer. As indicated above, developing countries constitute high percentages; they represent 86 % of the new cases and 88 % of the deaths [2].

In Sub-Saharan Africa the occurrence and mortality rates are amongst the highest in the world. Cervical cancer accounts for 34.5/100,000 of all cancers in women and a mortality rate of 25.3 per 100,000 in Eastern Africa. It is also reported that women in Sub-Saharan Africa lose more years to cervical cancer than any type of cancer [3].

Ethiopia also shares the high incidence of cervical cancer. According to the WHO estimates, about 4,648 cases (11.5 per 100,000) and 3,235 deaths are reported each year. Cervical cancer is also estimated to be the second most common cancer and the leading cause of cancer related deaths among Ethiopian women [4].

In addition, according to an unpublished report from Tikur Anbessa radiotherapy unit, women make up 70 % of the total cancer patients and from this number, cervical cancer patients are estimated to account for 30 %.

What’s more, based on the Cancer Registry of Addis Ababa City conducted in 2012–2013, cervical cancer is shown to be the second most common cancer in the city. In the data, cervical cancer cases accounted for 466 out of 4,106 of overall cancer cases in Addis Ababa (Unpublished, Addis Ababa Cancer Registry, 2013).

There are various social, cultural, economic and biological factors involved in the development of the disease. Based on multiple researches conducted, WHO established Human Papilloma Virus (HPV) as the leading cause for the development of cervical cancer in 1991 [5]. HPV is a very common infection which is transmitted through direct skin to skin contact of the genital areas. Though HPV is considered as the main cause of cervical cancer, multiple socioeconomic and cultural factors have been cited as contributing factors that highly increase the probability of exposure to HPV infection and the subsequent development of cervical cancer. These include; multiple sexual partners, early age at first sexual practice, early marriage, polygamy, multiple births (high parity), co-infection with other sexually transmitted diseases, tobacco and alcohol consumption [5].

Cervical cancer patients face multiple challenges in the process of diagnosis and treatment. The main challenges faced by patients include socioeconomic, cultural, health care based and psychological challenges. These challenges are documented as having an immense impact on the lives of women in various literatures [6–9]. As illustrated below, few researches have been conducted that assessed different dimensions of cervical cancer in Ethiopia, however, there has not been any study that has dealt with the variables examined in this research.

In un-published cross sectional survey conducted for a master’s thesis, Yehualashet Tadesse, assessed the factors that affect the diagnosis and treatment of cervical cancer in public health institution. He explored the awareness of health care providers of the disease and identified the available therapeutic and diagnostic infrastructure for the treatment of cervical cancer. The study surveyed 34 public health institutions (8 hospitals and 26 health centers). The outcome of the research showed the lack of awareness of the disease among the surveyed practitioners. Moreover, lack of equipment, proper documentation for diagnosis and treatment of cervical cancer were seen in the majority of the institutions.

Yifru Terefe and Asheber Gaym [10], focused on exploring the knowledge, attitude and practice of screening for cervical cancer among women reproductive health service clients in three referral hospitals. A cross sectional study was conducted on 276 reproductive clients attending emergency and regular outpatient departments. The results of the study showed an extensive lack of awareness of Pap smear testing among respondents compared to other developing countries particularly Sub-Saharan African countries. Moreover, the main source of information for women who have heard of pap smear were health care institutions which indicated the lack of utilization of other forms of information sources such as the media. What’s more, another study by Alemayehu Hailu and Damien Hailemaiam [11] in Tikur Anbesa Hospital measured the cost incurred by patients in the process of their treatment. The result of the study which included 227 patients in a cross-sectional survey revealed the economic burden associated with the treatment of cervical cancer. In the study, the average outpatient cost per patient for cervical cancer was found to be ETB 5, 905 ($407.2) while the outpatient cost for almost half of the respondent fell in a range between ETB 6,933 ($478) and ETB 1,359 ($93.7). Moreover, the average direct inpatient cost was found to be ETB 4,771 ($329) 74 % was spent on medical costs and 26 % on non-medical costs.

These studies explored important elements that are relevant for understanding different aspects of cervical cancer in Ethiopia. However, as indicated above the social, economic and cultural vulnerabilities and the challenges faced by women after their diagnosis have not yet been explored. Consequently, this work examined these variables in an attempt to fill this literature gap.

### Research questions

1. What factors increase women’s susceptibility to cervical cancer?
2. What are the particular problems women with cervical cancer face?

## Methods

### Study design

The study employed a mixed approach; it involved a descriptive cross –sectional quantitative and qualitative design. The qualitative design was used to obtain an in-depth look of the respective backgrounds, circumstances and challenges encountered by women in the study. The qualitative approach was employed as supplementary data source. It was used to illustrate the experiences of women prior to, and after their diagnosis and give the quantitative data context.

### Study area

The study was conducted in Tikur Anbessa specialized hospital which is located in Addis Ababa, the capital city of Ethiopia. Tikur Anbessa Hospital serves as the main government hospital in Ethiopia and accepts referrals from all over the nation. It was selected as the study site because it is the only central referral hospital in Ethiopia that has an oncology ward and that provides radiation therapy and chemotherapy treatments for cancer patients. The hospital has 560 inpatient beds and offers diagnosis and treatment for approximately 370,000- 400,000 patients per year. There are 201 physicians and 627 nurses in the hospital.

The oncology ward which has been serving as the main center for cancer patients was established in 1997. Recorded data indicate that the hospital has treated over 19,000 cancer patients to date. The cancer center currently has three oncologists, 4 Radiotherapists and 26 nurses. Further, the cancer center provides 16 beds for inpatient care.

The study was specifically carried out in the gynecology and radiotherapy departments of the hospital. The gynecology department deals with evaluation and surgical treatment, while the radiotherapy department exclusively deals with providing chemotherapy, radiotherapy and palliative care for cancer patients.

### Study participants

A total of 198 clinically diagnosed, subsequently confirmed, willing and physically able cervical cancer patients were enrolled in the study. Samples of 155 patients were taken from the radiotherapy department and 43 were taken from the gynecology department. In addition, 177 participants were outpatients while 21 were inpatients.

### Sampling technique

A Consecutive sampling technique was utilized to select the survey participants. This method was chosen for this study due to the unavailability of a sampling frame for the study population. Patients were approached as they came into the center individually, they were asked to participate in the study after a thorough explanation of the study aims. A total of 6 patients were excluded due to their physical inability (extreme fatigue) and another 3 participants who were in treatment for chemotherapy were excluded due to poor physical state at the time of data collection.

Ten cervical cancer patients were selected for the in-depth interview using a purposive sampling technique. In-depth interview participants were selected by the principal investigator.

### Sample size for quantitative data

The minimum sampling size was calculated using single proportion formula. Due to the unavailability of similar studies in relation to cervical cancer in Ethiopia. The sample size was calculated by using an unpublished master thesis conducted in Haiti in 2011 titled “Assessing women’s vulnerability to cervical cancer because of socio-demographic care access”. This study was chosen for the current research based on the similarity of the topic and variables under discussion. The study was a cross sectional study conducted to assess the socio- demographic and economic predicators among high risk group women for cervical cancer. Out of the five independent variables explored in the Haiti research namely: education, money, wealth, distance and residence; money was selected to calculate the prevalence (P) value for the current study. According to the findings in the Haiti study, 84.8 % of women responded that money represented a major constraint in getting medical help.

$$ n=\frac{Z^2\times P\left(1-P\right)}{d^2}=\frac{1.96^2\times 0.848\times 0.152}{0.05^2}=198 $$

Where: n = Sample size

z = Reliability Coefficient for 95 % confidence interval (1.96)

p = Prevalence of 0.848

d = Marginal Error (5 % = 0.05)

### Sampling size for qualitative data

As illustrated above, 10 participants were selected to obtain quantitative data. The qualitative data was mainly utilized to complement the quantitative data. Consequently, the information obtained from the selected number of study participants was deemed sufficient by the researcher.

### Research instruments

A structured, pre tested questionnaire was used for the quantitative part of the study. The questionnaire was initially prepared in English and translated to Amharic and finally translated back to English to confirm the validity of the translation.

For qualitative data collection, in-depth interview guide was employed. Questions related to socio- economic and cultural vulnerability and challenges faced by cervical cancer patients were thoroughly explored.

### Data collection

The data was collected from April 15 - May 15, 2013. The quantitative data collection and supervision was carried out by two nurses who had previous data collection experience. To ensure standardization and good quality of data, two days training was given on the questionnaire and the informed consent process. Supervision was undertaken by the researcher and supervisor throughout the data collection process. All submitted filled questionnaires were checked by the researcher for consistency. The questionnaire was piloted on five women who were attending care at the gynecology and radiotherapy departments in Tikur Anbessa Hospital. Piloting was done with data collectors to explore any difficulties that may occur in the actual data collection process, to observe the interaction of data collectors with the respective respondents, identify gaps in the questioning process and test the reliability and validity of the questionnaire. Corrections on the questions as well as overall data collection procedure were made accordingly after the pilot phase was completed. The questionnaires used for piloting were not included in the study analysis of the main study. Since most of the participants were illiterate the questions were read to them and they responded accordingly.

In-depth interviews were carried out with the selected cervical cancer patients. Every effort was made to make the participants feel as comfortable as possible. The researcher was able to secure a quiet room to conduct the interviews, this was of prime importance due to the sensitivity of information sought to be acquired. In this setting more informal conversation took place in which participants were able to share their feeling, beliefs, thoughts and experiences freely. To ensure the attainment of good data, theme based questions and probing were used. The prior was used to prepare a focused and thorough questioning and the latter was used to avoid subjective interpretation of the interviewees’ responses.

Field notes were used to capture all of the information; this was done with the full consent of the participants. The researcher has stored the paper version of the questionnaire in a storage place which is accessed solely by the researcher and the computer version of the data is protected by a password.

### Data analysis

The quantitative data was entered, cleared and analyzed using SPSS Version 19 for Windows. A descriptive analysis using scores, frequencies, percentages mean, median and range were calculated.

The qualitative data was transcribed and translated by the principal investigator. Then, analysis was conducted using thematic content analysis techniques. The qualitative data was analyzed manually using codes. The codes were merged into categories and the themes were determined based on the combination of similar categories.

### Ethical consideration

Ethical clearance was obtained from Addis Ababa University College of Health Science Institutional Review Board (IRB) in Tikir Anbessa on April 16, 2013.

To ensure full cooperation and understanding of the research aims by the participants, an information sheet and a consent form was prepared & administered by trained data collectors to each participant. The sheet contained information about the purpose of the study, confidentiality and the right of the respondents not to take part in the study. Participants signed the forms after all of the information was explained to them by the data collectors. Accordingly, the data collection process proceeded with a signed consent form from each participant.

### Limitation of the study

The data that was acquired from the questionnaire entirely depends on self-reported accounts of patients. Participants for the most part were asked to recall past events such as time duration for appointments, different costs they incurred and experiences they had related to year of marriage, last delivery, sexual behavior/ practices among others. This may create a recall bias which may result in inaccurate depiction of past events.

## Result and discussion

### Demographic characteristics

A total of 198 participants were surveyed. As illustrated in Table 1 the respondents were between the ages of 26 to 75 years with an average of 50 years. Only 4 (2 %) of the population were under 30 years old while the majority 104 (52.6 %) belonged to the 40–59 age group. The participants were from all the 9 regions and 2 city councils of Ethiopia. The majority were from the regions of Oromia 70 (35.4 %), Amhara 46 (23.2 %) and Addis Ababa 42 (21.2 %). SNNPR and Tigray followed with 15 (7.6 %) and 10 (5.1 %) respectively.

**Table 1:** Socio-demographic characteristics of respondents

Age group	N (%)
<30	4 (2.0)
30-39	35 (17.7)
40-49	54 (27.3)
50-59	50 (25.3)
60-69	45 (22.7)
>69	10 (5.1)
Current marital status	
Single	1 (0.5)
Married	101 (51.0)
Widowed	69 (34.8)
Separated	19 (9.6)
Divorced	8 (4.0)
Permanent residence (Region)	
Oromia	70 (35.4)
Amhara	46 (23.2)
Addis Ababa	42 (21.2)
SNNPR	15 (7.6)
Tigray	10 (5.1)
Harrari	6 (3.0)
Afar	3 (1.5)
Gambella	2 (1.0)
Somali	2 (1.0)
Benishangul-Gumuz	1 (0.5)
Dire-Dawa	1 (0.5)
Religion	
Christian Orthodox	123 (62.1)
Christian Protestant	29 (14.6)
Muslim	46 (23.2)
Occupation	
House Wife	78 (39.4)
Farmer	73 (36.9)
Government employed	13 (6.6)
Merchant (private business)	12 (6.1)
Employed in private for profit sector	9 (4.5)
Pensioner	8 (4.0)
Daily laborer	2 (1.0)
NGO employed	1 (0.5)
Unemployed	1 (0.5)
Temporary worker	1 (0.5)
Educational status	
Illiterate	103 (52.0)
Read and write only	29 (14.6)
Grade 1-4	21 (10.6
Grade 5-8	27 (13.6)
Grade 9-12	12 (6. 1)
College, University graduated	6 (3.0)
Number of children (parity) (*n* = 195); Mean = 5.3	
<=3	54 (27.7)
4-6	80 (41.0)
7-9	43 (22.1)
10+	18 (9.2)
Household monthly income in ETB (*n* = 180)	
<500	43 (23.9)
500 - 999.99	77 (42.8)
1,000 - 1,499.99	29 (16.1)
1,500 - 1,999.99	19 (10.6)
2,000 or more	12 (6.7)
Family size	
<=3	69 (34.8)
4--6	85 (42.9)
7--9	29 (14.7)
10+	15 (7.6)

In addition, more than half 101 (51 %) of the respondents were currently married, which was followed by those whose husbands were deceased 69 (34.8 %). Only one participant was single while the rest, 19 (9.6 %) and 8 (4 %) reported being separated and divorced respectively.

### Socio -economic vulnerabilities

In regards to the participants occupation, majority of the women were found to be housewives 78 (39.4 %) and farmers 73 (36.9 %). In regards to education, the majority 103 (52 %) were found to be illiterate. Those who could read and write, attended grades 1–4, and 5–8 were 29 (14.6 %), 21 (10.6 %), and 27 (13.6 %) respectively. The percentage of participants who attended secondary and higher education were very minimal. Only 6 (3 %) and 12 (6 %) of the population attended secondary and higher education respectively.

There has been a consistent association between low socio economic status as defined by education, income and occupation and cervical cancer in multiple researches [12–15]. Cervical Cancer is often referred to as a disease of “poor, uneducated and underserved women” [16]. Though the association made between socio-economic status and cervical cancer is not a direct association for the risk of cervical cancer, it has a significant implication for the exposure to HPV and development of cervical cancer. Though this association is not calculated in this study, it’s important to use previous studies which have made this association to understand the findings within the current research. Several factors are involved in making this association; a women’s level of education, income, and occupation are generally thought to influence the level of decision making and exposure to information women have.

Women with low education and income are more likely to have less awareness of cervical cancer and preventive mechanisms which consequently may lead to inadequate screening and gynecological follow up [17].

Additionally, women who are uneducated, unemployed or underemployed are more likely to be dependent on their husbands economically. These issues were clearly observed during the in-depth interview with the participants. Six of the women in the interviews who were farmers, reported having more of a supportive role in income generating activities rather than a leading role. Most of the respondents stated that their main responsibilities lie more on household activities such as child rearing and household chores rather than income generating activities. The interrelationship of these circumstances with the constraining cultural roles and norms prevalent in the Ethiopian context places women in vulnerable position.

As shown on Table 1, the size of household members ranged from 1 to 13. The average household size was 4.8 persons. (77.7 %) of the population live with up to 5 people. In regards to household income, 149 (82.8 %) of the population earned less than 1,500 ETB (78.9 USD). The mean and median value of household income was 879.9 ETB (46.3 USD) and 675 ETB (35.5 USD) respectively. The maximum reported household income was 3,700 ETB (194.7 USD) while the minimum was 100 ETB (5.2 USD). The majority of participants reported having only one income earner to support their entire family.

This shows that majority of the study population were living in poverty. According the World Bank report of 2005, those who earn less than 1.25 dollar per day were considered to be in the poverty line [18]. In the current study, the approximate monthly income for the majority (77 %) of the households was established to be 1.54 dollar per day for approximately 5 household size (using an exchange rate of 19 birr per dollar). This implies that these households are living below the poverty line. In these circumstances, women’s tendencies and abilities to prioritize their health is largely compromised. One respondent’s story clearly illustrates this aspect. Patient1 is 45 years old women and a mother of five who recently separated with her husband due to her illness. She is a farmer and sole provider for her family. Since she lives in a remote town outside of Dessie, she has to travel one day by car to get to a health care facility;

*“My disease initially started with abdominal pain and then it slowly progressed to a fluid discharge. Despite these symptoms I couldn’t go to the city to seek care because I had no one to provide and care for my children”*.

Socio-economic and cultural factors are factors that overlap in influencing the health seeking behavior of women. Women might usually resort to faith based institutions or traditional healing before seeking medical treatment. In addition, routine checkups and visits to health care institutions by women is not a norm in developing countries especially Africa. Though socio-cultural factors play an important role in influencing the health seeking behavior of women, economical factor (the ability to afford treatment and care) is also a crucial factor that affects the health seeking tendency of women. The data shows that majority of women have to share household earnings with up to 5 people. This leads to the inability of women to access good health care in the present study; women by and large were found to have a lower socio-economic status which highly compromises their ability to access to good health care.

### Cultural vulnerabilities

#### Early marriage and sexual exposure

The age at first marriage ranged from 8 to 31 years with an average age of 16 years. As can be seen from Table 2, 109 (55.9 %) of the population were married before the age of 16 and the greater majority 180 (92.3 %) were married before the age of 21. In regards to the mode of marriage, the vast majority 160 (81.2 %) were married by family arrangement while only 31(15.7 %) were married based on their own choice.

**Table 2:** Mode of marriage, age at first marriage, sexual intercourse, number of lifetime sexual partners of the respondents

Characteristics	n (%)
Mode of marriage (*n* = 197)	
Abduction	6 (3)
Family arrangement	160 (81.2)
Own choice	31 (15.7)
Age at first marriage (*n* = 195)	
<=12	22 (11.3)
13-16	87 (44.6)
17-20	71 (36.4)
21+	15 (7.7)
Age at first sexual intercourse (*n* = 195)	
<=12	14 (7.2)
13-16	100 (51.3)
17-20	74 (37.9)
21+	7 (3.6)
Number of life time sexual partners of women	
1	103 (52.3)
2	65 (33.0)
3 or more	29 (14.7)

These figures clearly show the imbalance of the power dynamics. Family and society by and large are said to set the context for meanings, values and norms related to the appropriate behavior expected and allowed for women. Adherence to these supposed norms and cultures deemed “appropriate behavior” for women suppresses their right to choose. When women are given off for marriage at such a young age, their exposure to education and information, capabilities to sustain themselves economically and their ability to attend to their health becomes compromised. These issues tie in to the exposure of women to several negative health outcomes one of which is cervical cancer.

In regards to the number of life time sexual partners as seen in Table 2, 103 (52.3 %) of the participants had one sexual partner (their husbands). Furthermore, 65 (33 %) of the participants stated they had two sexual partners while only 29 (14.7 %) had three or more sexual partners. According to various studies [15, 19, 20] conducted early age at first sexual intercourse and having multiple sexual partners are considered as factors that make women susceptible to the exposure of HPV. Early age at first sexual intercourse predisposes women to HPV infection due to the lack of development of the cervix. In addition, having several sexual partners also puts women at a higher risk of acquiring the HPV infection. However, in the current study several sexual partners were not observed for the majority of the patients in the study. The majority 103 (52 %) of respondents had only one sexual partner (their husbands) and the majority of women 109 (55.9 %) were married before the age of 16 while 180 (92.3 %) were married before the age of 21 which indicates early age of marriage. Moreover, the average age at first marriage and the average (mean) age at first sexual intercourse were found to be (16.5 and 16.6 years respectively) which is in the same age range. From these facts it can be inferred that the probable cause of HPV infection for the majority of the current study population was early age at sexual intercourse with their husbands.

### Polygamy

As shown in Table 3, over a third, 53 (26.9 %), of the participants were found to be in polygamous marriages. The apparent previous sexual experiences of husbands were also observed. Though the majority 103 (52.3 %) of the women’s first sexual encounter was with their husbands, the majority, 104(57.8 %), of the participant’s husband’s had previous wives before marrying them. 56 (53.8 %) of the study participants husbands had one wife while 48 (46.1 %) had two or more wives. In addition, out of the 53 women who were in polygamous marriage, (91.3 %) stated that their husband had a wife or wives before being married to them.

**Table 3:** Magnitude of polygamous marriage among patients with cervical cancer

Characteristics	N (%)
Prior marriage of husbands (*n* = 180)	
Yes	104 (57.8)
No	76 (42.2)
Number of Wives Of Husbands (*n* = 104)	
1	56 (53.8)
2	24 (23.1)
3	16 (15.4)
4	4 (3.8)
5	4 (3.8)
Respondents in Polygamous Marriage (*n* = 197)	
Yes	53 (26.9)
No	144 (73.1)
Number of Wives of Husbands (*n* = 53)	
One	34 (64.2)
Two	13 (24.5)
Three	4 (7.5)
Four	2 (3.8)

As clearly shown in the various studies conducted, such as the ones Mali and Algiers [15, 19] having multiple life time sexual partners was a significant factor for exposure to HPV infection. Many factors have been shown to play a role for the development of cervical cancer, however, the exposure to HPV infection through sexual intercourse is the necessary factor that primarily put women at a higher risk for cervical cancer. In line with this fact, given their age at first sexual intercourse and the relative previous sexual experiences of their husbands, it is highly probable that the male factor played a significant role in the transmission of HPV for the majority of cases.

### Challenges faced by patients in the course of diagnosis and treatment

#### Challenges related to cost of treatment

The economical challenge of cervical cancer patients and their respective families is immense. As shown in the previous section in Table 1 the household income of the majority of the respondents 149 (82.8 %) is less than 1,500 ETB (78.9 USD) per month which is a small amount taking the large family size the households support into consideration. The majority of participants reported having only one income earner to support an entire family of 4.89 individuals on average. These factors tie in to the enormity of the economical challenge placed on cancer patients and their families.

For those who are currently in treatment (116), the mean expenditure for diagnostic tests and treatment was 4,896 ETB (257.6 USD) and the mean expenditure for food, transport and lodging was 2,304 ETB (121.2 USD).

### Direct costs

Table 4 illustrates the estimated costs for test, treatment, lodging, transportation, food, and source of major financer. In regards to the expenses related to tests and treatments, out of the 183 patients who were able to estimate the cost of tests and treatment, the majority 76 (65.5 %) reported spending from 1000–7000 ETB (52.6-368.4 USD) while only 6 (5.2 %) reported spending less than 1,000 ETB (52.6 USD). 98 (51 %), of the respondents cannot afford to pay for treatments by themselves, consequently, they reported relying on family support for their expenses while, the second majority 51 (26.7 %) stated incurring the cost by themselves.

**Table 4:** Estimated expenses for test, treatment, lodging, transportation, food, and source of major financer

	Number	Percent
Source of finance (*n* = 191)		
Myself	51	26.7
Family members	98	51.3
My work institution	2	1.0
Hospital/free service	34	17.8
NGO/other institution	6	3.1
Expenditure for tests and treatments (*n* = 116)		
<1000	6	5.2
1,000 - 2,999	36	31.0
3,000 - 4,999	25	21.6
5,000 - 6,999	15	12.9
7,000 - 8,900	13	11.2
9,000 or more	21	18.1
Expenditure for transportation, food and lodging (*n* = 118)		
<1000	25	21.2
1,000 - 2,999	54	45.8
3,000 - 4,999	20	16.9
5,000 - 6,999	14	11.9
7,000 - 8,900	3	2.5
9,000 or more	2	1.7

### Indirect costs

As shown in Table 5, 140 (70.7 %) of the participants were from other regions out of the city of Addis Ababa. The majority in the study participants were staying in lodgings 85 (61.2 %) and with family members 40 (28.8 %) while attending care. 88 (74.6 %) reported spending from 1000–7000 ETB (52.6-368.4 USD) on costs related to lodgings, food, and transportation while only 25 (21.2 %) reported spending less than 1,000 ETB (52.6 USD).

**Table 5:** Indirect Costs of Participants

	Number	Percent
Number of patients attending care by travelling from other parts of the country (*n* = 198)		
Yes	140	70.7
No	58	29.3
Place of stay for medical follow up (*n* = 140)		
Family Members	40	28.8
Lodging	85	61.2
Going back and forth	11	7.9
Other (Cancer Care, Hospice Ethiopia )	4	2.2

When these cost related challenges were further explored through the in-depth interview, women shared their respective challenges. Due to their illness, most of the women stated being physically incapacitated and not being able to work which placed an enormous burden on their husbands and respective family members. The women also shared the different mechanisms they used to cope with the expenses. Apart from relying on family members, some of the respondents relied on begging in the streets & selling household goods to pay for their medical expenses and accommodation. The economic burden placed on family members and patients were clearly observed in one of the respondent’s story.

Patient 4 is from *Wellele* in *Gurage* Zone. She is a widow and a mother of five. All of her children are under 10 years old and she is the only provider for her family. She makes her living by cutting *kocho* which is a stable food in Gurage zone;

*“I don’t have any money to pay for my treatment. So my little brother begs on the streets to get money to sustain us. We are facing problems affording the lodging and food to eat. Since we are solely dependent on the money we get from begging, there is a lot of uncertainty. We might eat one day, but not another”.*

The expenses related to cervical cancer can also have the potential to break up family members. Since majority of the patients live in poverty, their living circumstances are usually hand to mouth. The additional expenses of the disease on their already fragile circumstances can create problems within their household dynamics. This was illustrated by five of the respondents in the in-depth interview; they reported having constant clashes with their husbands due to the cost they incurred after their diagnosis. One respondent Age 43, from *Wollo* illustrated this aspect;

*“Due to the long waiting lists, I used to come back and forth from my home town without getting any treatment. I used to argue with my husband because he always accused me of wasting money by going back and forth without getting any better”*.

Women have a productive as well as a reproductive role. Their active participation is very essential for a well-run household. Thus, the incapacitation of women due to their ailment has a significant negative impact for the overall family dynamics.

### Psycho-social challenges

The psychological challenges of women were explored in a qualitative inquiry. Similar to various studies conducted in this area [6,21], patients reported facing tremendous challenges in regards to the side effects from treatments. The side effects encountered included; fatigue, lesions, urinary urgency & leakage, diarrhea, loss of appetite, nausea and fistula. The smell of the liquid discharge caused by the disease was cited as another major challenge. As reported by the women, these side effects and the disease outcome had seriously affected their social interaction. Many stated feeling ashamed to be around people during the times they experienced these side effects. One in-depth interview participant’s story clearly shows this aspect;

*“The smell of the liquid discharge is highly potent. I don’t want to be near people when it happens. When I am at home, I usually stay in a room by myself and don’t let anyone come near me. And when I come to the hospital to attend care, I usually isolate myself from other patients and sit alone because the smell embarrasses me”*.

Additionally, the disease impacted the relationship they had with their husbands. Due to the nature of the illness, women have to go through several treatments that have the potential to affect the sexual and reproductive desire and abilities of women; pain during intercourse, fear of recurrence, lack of sexual arousal and partner dissatisfaction have been identified in the in-depth interview. Though these kinds of sexual dysfunctions have been reported in previous researches [22, 23] as causing moderate to severe distress in marriages, in the current study, only two participants reported facing severe distress in their marriages. In the in-depth interview, two participants reported separating with their husbands after their diagnosis. One of the participants reported ending her marriage with her husband because she wanted to concentrate on her health and treatment, while the other respondent reported being separated with her husband due to her unwillingness to continue sexual intercourse after her diagnosis.

Fear of recurrence and lack of interest for sexual intercourse were the other major themes that run across most of the patients. All of the patients revealed a desire to concentrate on the issue of survival and getting better. Patients felt that if they resumed sexual intercourse the disease would come back. Thus, when the subject of sexuality and marital problems were raised, all patients expressed their desire to concentrate on their health. Though marital problems were not observed in the majority of the respondents, the psychological impacts are nonetheless evident. The patients reported feeling constant anxiety related to recurrence.

A hierarchical relationship between patients and health care practitioners has also been widely seen. As shown in the figures within this research, most of the participants are from a lower socio-economic background with very little education. This has a significant implication on the level of understanding these women have in regards to general health issues and cancer in particular. When asked about the extent of the knowledge they had about their current illness, most of them asserted knowing very little and some reported not even knowing about their treatments. Furthermore, patients reported not being told about their treatment options, side effects and even about their disease by their doctors. The lack of knowledge of their disease created a sense of constant fear and anxiety of death and recurrence. The overwhelming number of patients and the ill-equipped nature of the hospital can be one of the major reasons for the hierarchical relationships observed. Since doctors have to see hundreds of patients a week, having in-depth conversation with all patients might not be feasible. Nonetheless, cancer patients are very vulnerable, appropriate counseling service is essential to help in the understanding and coping with their illness.

The negative societal attitude has also been cited as a major source of distress for patients in the study. They stated hiding their disease from neighbors and extended family members because of negative social perception about the disease and its outcomes.

Those who have revealed their illness reported being told that they are not going to survive and overall receiving negative attitudes. On top of the severity of the illness, being subjected to these attitudes created an immense psychological impact on these women.

### Health care based challenges

#### Practitioner and system delays

In the health care system, patients have to pass through different levels of care (primary, secondary and tertiary) before getting their final diagnosis and treatment; due to this fact patients endure several practitioner and system delays [24, 25]. Practitioner and system delay among others were evident in the present study.

Patients lost a lot of time as a result of misdiagnosis. As shown in Table 6, the majority of the patients had evident initial gynecological symptoms; unusual vaginal bleeding 172 (38.5 %) and unusual heavy discharge 132 (29.5 %). Despite these symptoms however, three fourth of them were misdiagnosed with other illnesses. This report of misdiagnosis was further substantiated by an in-depth interview, out of the total ten patients interviewed, all but one of the respondents were misdiagnosed with other illness upon their visit to health care practitioners. Most of them were misdiagnosed with diseases such as STD’s, gastritis and kidney disease. This created a significant delay for the patients. One in-depth interview participant from Debre Zeit, age 70, illustrates this aspect clearly;

**Table 6:** Initial symptoms, number of health facilities visited, time span of referral, stage at diagnosis, and type of treatment received

Characteristics	Number	Percent
Initial symptoms encountered		
Unusual vaginal bleeding	172	38.5
Unusual heavy discharge	132	29.5
Pelvic pain	67	15.0
Pain during urination	39	8.7
Bleeding after sexual intercourse	22	4.9
Other (Back pain, bone pain, leg pain…)	15	3.4
Number of health facilities visited before being referred to Tikur Anbessa (*n* = 198)		
1	59	29.8
2	83	41.9
3	36	18.2
4	10	5.1
5	7	3.5
6 or more	3	1.5
Time span of referral to Tikur Anbessa from first health care visit (*n* = 196)		
Less than 1 month	38	19.4
1 - 2 month	63	32.1
3 - 5 months	52	26.5
6 months - 1 year	16	8.2
1 year or more	27	13.8
Time span from referral to Tikur Anbessa and receiving a diagnosis (*n* = 175)		
Less than 1 month	16	9.1
1 - 2 month	112	64.0
3 - 5 months	42	24.0
6 months - 1 year	4	2.3
1 year or more	1	0.6
Diagnosed in other Institutions 23		
Time interval from diagnosis to treatment in Tikur Anbessa (*n* = 128)		
Less than 1 month	5	3.9
1 - 2 months	30	23.4
3 - 6 months	79	61.7
More than 6 months	14	10.9
Type of treatment received (*n* = 128)		
Surgery	9	7.0
Chemotherapy	3	2.3
Radiotherapy	80	62.5
Surgery and Radiotherapy	3	2.3
Radiotherapy and Chemo therapy	29	22.7
A combination of 1, 2 and 3	4	3.1
Stage of cervical cancer at diagnosis		
Stage III	59	46.0
Stage II	49	38.0
Stage IV	17	13.0
Stage I	4	3.0

*“At first, I had heavy vaginal bleeding. I was told I had a urinary tract infection on my first health care visit. And when my symptoms got worse after taking the prescribed medication, I went to another doctor, where I was told that I had kidney stones. I visited four other health care institutions in six months, all of which misdiagnosed me with other diseases. After a lot of pain and agony, I was finally diagnosed with my disease upon my fifth health care institution visit”.*

Patients also encountered significant delay in the referral process. As shown in Table 6 the majority 178 (89.9 %) had to visit up to three health care institutions and 20 (10.1 %) visited 4 or more health care institutions before being referred to Tikur Anbessa. Moreover, on average, participants spent 5 months in visiting other health care institutions until they finally came to Tikur Anbessa where they were diagnosed. The majority (175) of them despite visits to several other institutions were diagnosed at Tikur Anbessa.

After coming to Tikur Anbessa, patients had to go through additional waiting time for tests and respective treatments. The majority 112 (64 %) and 42 (24 %) of the patients had to wait additional 1–2 months and 3–5 months respectively for their diagnosis and another four months on average to get treatments in Tikur Anbessa. After being referred to the cancer treatment center, patients have to wait for an additional 2–6 months to be seen by a doctor. Thus, overall, majority of patients have to wait 3 to 7 months at a minimum to get treatment after coming to Tikur Anbessa.

The delay in Tikur Anbessa Hospital has been attributed to different factors. Since Tikur Anbessa is the only cancer center in the country, significant burden is placed on the institution. It accepts referrals from all parts of the country for all types of cancers. According to the Head of Oncology at Tikur Anbessa Hospital, the center is estimated to treat over 2,500 patients a year. In spite of this fact however, it is significantly short staffed and under equipped. There are only 3 oncologists for all cancer patients and 4 Radiotherapists. Moreover, there are only two radiotherapy machines, but since the machines break down regularly only one machine is available for treatment of all patients. The interview with the head of oncology revealed that, at one point both of the machines broke down and was not fixed for over eight months which caused a lot of delays and suffering for patients. The prolonged maintenance period was attributed to high maintenance expenses and lack of skilled professionals to carry out the needed repairs.

As most patients arrive to the center at a late stage of their cancer, the treatment for most of the cases as seen in Table 6 is radiotherapy treatment. According to the interviews with the senior Radiotherapists at Tikur Anbessa Hospital, patients who are in stages three and four are treated as palliative cases in which radiotherapy is given as a mechanism for pain relief. Additionally, the data revealed that, out of the study population, only 12 (9.3 %) did not require radiotherapy treatment. This indicates the importance of the radiotherapy treatment in the overall care for cervical cancer in center. Thus, the impact of the breakdown of the machines is immense.

In the current study, 59 (46 %) and 49 (38 %), as shown in Table 6, are in stage II and III respectively, which is considered an advanced stage. Though this factor cannot solely be associated with system and practitioner delay, these factors have been shown to have a significant impact on the progress of the disease. When this issue was further explored in the interviews with various medical practitioners in Tikur Anbessa Hospital, it was reported that those who arrive at early stage usually advance to late stage due to the long waiting times. An in-depth interview with a patient from Arsi, age 49, illustrates the situation clearly;

*“After being diagnosed, I was told that I needed to have surgery performed to remove the tumor right away. Due to the long waiting list however, I was told I had to wait for 4 months to have the surgery. After waiting for 4 months, I was told that I still needed to wait another two months. I was finally admitted to the hospital and underwent a re-evaluation process. After the re-evaluation procedure, the doctors informed me that my tumor had grown and that they couldn’t operate. Thus, I was referred to the Radiology center where I had to wait another 3 months for radiation treatment”.*

This is the story of many women with cervical cancer in Ethiopia. Those who were lucky enough to be diagnosed early are not able to be treated urgently due to these delays. Many lives are lost that could have been saved because of practitioner and system delay.

## Conclusion

This study uncovered numerous vulnerabilities of women to cervical cancer and challenges faced by cervical cancer patients in Ethiopia. In the study it was revealed that cervical cancer is a disproportionately affecting women that have a low socio- economic status. Primary and secondary prevention mechanisms such as awareness creation, vaccination and screening targeting particularly the poor segment of the population should be widely initiated and strengthened. What’s more, expansion of cancer treatment sites in different parts of the country will aid in decreasing the burden placed on the current center and it will also help in averting the challenges faced by patients in terms of cost and accommodation. Furthermore, cancer patients are very vulnerable, appropriate counseling and psychosocial support is essential to help in the understanding and coping with their illness.

## Abbreviation

WHO: World Health Organization
HPV: Human papilloma virus
SPSS: Statistical package for the social sciences
ETB: Ethiopian birr
USD: United States Dollar
